# Sources of cigarettes for youth smokers in Malaysia: Findings from the National Health and Morbidity Survey (NHMS) 2022: Adolescents Health Survey (AHS)

**DOI:** 10.18332/tid/201987

**Published:** 2025-03-24

**Authors:** Kuang Hock Lim, Yoon Ling Cheong, Kuang Kuay Lim, Jia Hui Lim, Hamizatul Akmal Abdul Hamid, Mohd Ruhaizie Riyadzi, Sumarni Mohd Ghazali, Chee Cheong Kee, Cheah Yong Kang, Chong Shao Hui, Ali Aman Marine, Mohd Hazilas Mat Hashim, Hui Li Lim

**Affiliations:** 1 Institute for Medical Research, National Institutes of Health, Ministry of Health Malaysia, Shah Alam, Malaysia; 2 Institute for Public Health, National Institutes of Health, Ministry of Health Malaysia, Shah Alam, Malaysia; 3 School of Pharmacy, Faculty of Health and Medical Sciences, Taylor’s University, Subang Jaya, Malaysia; 4 Biostatistics and Data Repository Sector, National Institutes of Health, Shah Alam, Malaysia; 5 School of Economics, Finance and Banking, University Utara Malaysia, Sintok, Malaysia; 6 Faculty of Medicine and Health Sciences, University Putra Malaysia, Serdang, Malaysia; 7 Institute for Clinical Research, National Institutes of Health, Ministry of Health Malaysia, Shah Alam, Malaysia

**Keywords:** source of cigarettes, school adolescents, frequent smokers, commercial source, NHMS: AHS

## Abstract

**INTRODUCTION:**

Developing effective intervention programs to lower adolescent smoking requires a thorough understanding of the sources and methods of youth tobacco product acquisition. This study aimed to identify the sources of cigarettes and related variables among adolescent smokers in Malaysian schools using the latest national data from the National Health and Morbidity Survey: Adolescents Health (NHMS: AHS) 2022.

**METHODS:**

We conducted the NHMS 2022: AHS to obtain a representative sample of school-age teenagers via a cross-sectional study design and a multi-stage sampling approach. We selected 1934 school-going adolescents aged 13–17 years who have smoked at least once in the previous 30 days from a total of 33523 respondents in the study. Data were collected from the participants using a pre-validated self-administered questionnaire. The analysis involved calculating adjusted odds ratios (AORs) with 95% confidence intervals (95% CIs). Furthermore, we examined potential two-way interactions between the independent variables.

**RESULTS:**

The study found that 6.2% (95% CI: 5.9–6.6) of teenagers in schools are currently smoking, with a notably higher percentage of male to female current smokers (10.8% vs 1.6%). Approximately 23.1% of current smokers are frequent smokers. Almost three-quarters of current smokers obtained their cigarettes from fixed premises (38.9%), and that friends (34.9%) were the primary sources of cigarettes among adolescents. The data show that more than half (59.7%, 95% CI: 57.0–62.4) of current smokers obtained cigarettes from commercial sources.

**CONCLUSIONS:**

The study found that a notably more significant proportion of adolescent smokers obtained their cigarettes from commercial vendors compared to their friends. These finding implies that increased law enforcement and health promotion programs are needed to lower the incidence of adolescent smoking in Malaysia.

## INTRODUCTION

Smoking is the leading preventable cause of illness and death in Malaysia^1^. To address this, the Malaysian government has implemented comprehensive measures, including expanding smoke-free areas^2^, increasing tobacco prices, enhancing health promotion, and providing more smoking cessation facilities^3^. The legislation bans tobacco sales to minors and imposes a minimum cigarette price^4^. These actions align with the World Health Organization (WHO) Framework Convention on Tobacco Control’s (FCTC) recommendations to reduce tobacco access for adolescents^5^, recognizing that most adult smokers begin the habit before the age of 21 years^6,7^. Adolescent smoking, driven by abstract thinking and a sense of invincibility^8^, significantly increases the likelihood of adult smoking due to nicotine addiction^6,9^. Early smoking initiation raises the risk of smoking-related diseases and mortality^6^. Reducing adolescent smoking is crucial for lowering adult smoking rates and improving public health in Malaysia.

Efforts to prevent adolescents from acquiring tobacco products have involved educational initiatives urging them to make informed and sensible choices, particularly when it comes to their behavior, such as smoking^10^. Furthermore, the reduced accessibility of cigarettes to young individuals has indirectly resulted in a decrease in the frequency of their smoking and a decreased willingness to share their cigarettes^11^. These measures have been identified as one of the effective measures to reduce the incidence of adolescent smoking as demonstrated by various research, including those focused on lowering smoking initiation among adolescents^12,13^, as well as reducing the transition from occasional smoking to regular smoking^12^.

Research has indicated that adolescents commonly acquire tobacco products from their acquaintances and relatives. Moreover, epidemiological studies have shown that older teenagers^7,13-16^, male smokers^7,14^, and frequent smokers (who smoke more than 20 cigarettes or more in a day)^7^ are more likely to obtain cigarettes from commercial stores. The scenario is the opposite with young female smokers^14,16^. Likewise, it is more prevalent for occasional smokers – regardless of their gender – to obtain tobacco from their peers and other adults^14,16^.

Only a few studies have been conducted in Malaysia on the source of cigarettes among youth, in which three small-scale studies were focused on the source of cigarettes among adolescents, namely in Kota Tinggi in Johor^17^, Petaling District in Selangor^18^, and Kota Bharu in Kelantan^19^, Malaysia. Only one national study was carried out in 2016 to investigate the source of cigarettes among school-going adolescents aged 11–17 years in Malaysia. The study revealed that more than half of the current smokers obtained their cigarettes from commercial sources, and it is more prevalent among females aged 16–17 years and among frequent smokers (who smoke more than 20 days in a month)^7^. However, the evidence is more than five years old, and the dynamics of adolescent smoking and the source of cigarettes among Malaysian youth might have changed. Therefore, current information regarding how Malaysian adolescent smokers obtain cigarettes is crucial. These results will assist in identifying gaps in the current legal system and law enforcement efforts to limit adolescent access to smoke and are in line with the suggestion of WHO FCTC which urges the member countries to frequently monitor the smoking patterns among adolescents^5^. The current study describes the sources of cigarettes and associated factors using the latest National Health and Morbidity Survey 2022: Adolescent Health Survey.

## METHODS

### Study design and sampling

We conducted the NHMS: AHS 2022 nationwide from June to July 2022. A cross-sectional study design and a multi-stage sampling method were used to select a representative sample of secondary school-going adolescents using the sampling frame of 2021, including both private and public secondary schools under the purview of the Ministry of Education and the Ministry of Rural and Regional Development (MARA) in Malaysia^20^. The initial step of sampling is stratifying each state in Malaysia. Subsequently, each state is divided into urban and rural areas. Proportionate-to-size sampling was used to select each state’s primary sampling units (schools). We choose three to nine classes from each selected school via systematic random sampling. All students in the selected classes were invited to participate in the study. The Ministry of Education, Malaysia, approved the study’s protocol and the Medical Research Ethics Committee (MREC), Ministry of Health Malaysia, granted the ethical approval for the study (NMRR-21-157-28561; Date: 8 June 2021).

The sample size for this study is determined by employing a single proportion formula to calculate the objectives of each module. This proportion was obtained from the previous NHMS: AHS 2017. The largest sample size obtained from the calculation of the respective module was used for the study. The required sample size is 36000 after considering the design effect of two and estimating a non-response rate of 20% for the national level. Therefore, 2250 adolescent respondents are required for each state.

### Ethics

The surveys employed the active consent approach to acquire approval from the respondents’ parents or guardians. The parents/guardians of the selected respondents were provided with the consent form and a letter from the school administration. The letter explained the study’s objectives in detail, and the participation was voluntary. The anonymity of the respondents will be ensured and the data obtained will only be used for research purposes. The parents or guardians who consented for their children to participate in the study returned the completed form to the school administration.

### Measures

The National Health and Morbidity Survey: Adolescent Health Survey (NHMS: AHS) 2022 instrument was adopted from NHMS: AHS 2017. The questionnaire was pre-tested in selected schools in Kuala Lumpur to establish face validity and ensure that the assessment items were appropriate for the local sociocultural context. A minor modification was made based on the feedback from the pre-test session.

Data collection was conducted in the area provided by the school administration. No teachers or school staff attended the data collection session, and only the respondents who obtained approval were allowed to participate in the study. Before data collection, research team members briefed the participants. They explained the objectives of the survey, the contents of the questionnaire, the participant’s right to refuse to answer any question, and the confidentiality of the information they provided. Participants must sign additional consent forms and the parent/guardian consent. Research team members assist respondents who require clarification on the items in the questionnaire or have any inquiries on the content of the questionnaire.

### Dependent and independent variables

Only respondents <18 years who smoked at least one day during the last 30 days (i.e. current smokers), even one or two puffs, were included in the analysis; students aged ≥18 years were excluded because they were of legal age to purchase tobacco. The dependent variable in this study was measured by the item: ‘The last time you smoked cigarettes during the past 30 days, how did you get them?’^7^. The respondents who answered ‘I bought a cigarette from a retail shop/market/supermarket/24-hour store/petrol station (fixed premises)’ or ‘I bought a cigarette from a roadside stall/kiosk/vendor/night market (non-fixed premises)’ or ‘I bought a cigarette from a food store’ or ‘I bought a cigarette from an online selling portal or through social media’, were classified as having obtained a cigarette from the commercial source. At the same time, those smokers who chose ‘I got a cigarette from a friend’ or ‘I got a cigarette from a family member’, were classified as obtaining a cigarette from a ‘social source’. The respondents who answered ‘other ways’ were excluded from the analysis.

Independent variables in the study were gender, , ethnicity (Malay, Chinese, Indian, Bumiputra Sabah, Bumiputra Sarawak), age group (13–15; 16–17 years), type of smoker (frequent smoker: smoked 20 days or more during the last 30 days; infrequent smoker: smoked less than 20 days in the previous 30 days)^7^, parental smoking status (yes,no) and parental marital status (married vs widow/widower/divorce/separated).

### Data management and analysis

The data were cleaned, and the sample was a weighted sample based on the survey’s sampling design, response rate, and population characteristics to ensure valid population estimates from the analysis. We calculated the estimated population, percentage, and 95% confidence interval based on the weighted sample. The demographic features of the respondents are shown using descriptive statistics. The relationship between the ‘source of cigarettes’ and all categorical independent variables was ascertained using chi-squared analysis. Variables with p≤0.25 were included in the multivariable logistic regression (MLR) to identify the factors related to the sources of cigarettes. MLR was reported as AOR and 95% CI. The final model was tested for all possible two-way interactions (multiplicative interaction) between the independent variables to ensure no modification effect between the independent variables All statistical analysis and interaction analysis were run at a 95% confidence interval using the Complex sample analysis of SPSS statistical software version 22.

## RESULTS

The study found that 6.2% (95% CI: 5.9–6.6) of teenagers in schools are currently smoking, with a notably higher percentage of male to female current smokers more significant than 6 to 1 (10.8% vs 1.6%). The study also revealed a higher prevalence of smoking among Bumiputra Sarawak adolescents (14.5%, 95% CI: 12.5–16.8), followed by other ethnic adolescents (10.5%). Approximately 23.1% of current smokers are frequent smokers. In addition, the prevalence of smokers aged 13–15 years is 3% lower than their counterparts aged 16–17 years (Table 1).

**Table 1:** Sociodemographic characteristics of school going adolescents (current smokers) who participated in the National Health and Morbidity Survey 2022: Adolescent Health Survey (NHMS 2022: AHS) (N=1934)

*Characteristics*	*Estimated population*	*Sample*	*% (95% CI)*
**Gender**			
Male	110792	1664	10.8 (10.2–11.5)
Female	16720	270	1.6 (1.4–1.9)
**Ethnicity**			
Malay	85307	1370	6.6 (6.2–7.0)
Chinese	8096	102	2.2 (1.7–2.9)
Indian	4934	65	4.1 (3.1–5.5)
Bumiputra Sabah	9165	154	8.0 (6.5–9.7)
Bumiputra Sarawak	15236	161	14.5 (12.5–16.8)
Other	4273	82	10.5 (8.0–13.7)
**Age** (years)			
13–15	65708	931	5.1 (4.7–5.5)
16–17	61804	1003	8.1 (7.5–8.8)
**Parental marital status**			
Married	96987	1467	5.7 (5.3–6.0)
Separated	26054	401	8.7 (7.9–9.9)
**Parental smoking**			
Yes	73226	1081	8.7 (9.1–9.3)
No	41632	852	3.8 (3.5–4.2)

The estimated population and % (95% CI) were calculated based on a weighted sample based on survey’s sampling design, response rate, and population characteristics to ensure valid population estimates from the analysis.

Table 2 shows that almost three-quarters of current smokers obtained their cigarettes from fixed premises (38.9%), and friends (34.9%) were the primary sources of cigarettes among adolescents. Meanwhile, 3.8%, 4.1%, and 5.1% obtained their cigarettes from the food stores, online, and from family members.

**Table 2:** Source of cigarettes among school going adolescents (current smokers) who participated in the National Health and Morbidity Survey 2022: Adolescent Health Survey (NHMS 2022: AHS) (N=1934)

*Source*	*Estimated population*	*Sample*	*%*	*95% CI*
Purchase from a fixed premise	50168	743	38.9	36.2–41.6
Purchase from non-fixed premise	17075	280	13.2	11.6–15.1
Buying from a food store	4918	81	3.8	3.0–4.9
From on-line	5287	78	4.1	3.1–5.4
From friends	45050	662	34.9	32.3–37.6
Family members	6615	106	5.1	4.1–6.4

The estimated population and % (95% CI) were calculated based on a weighted sample based on survey’s sampling design, response rate, and population characteristics to ensure valid population estimates from the analysis.

The data show that more than half (59.7%, 95% CI: 57.0–62.4) of current smokers obtained cigarettes from commercial sources. Among young males, the proportion was significantly higher at 61.6% (95% CI: 59.6–64.5) than young females at 48.5% (95% CI: 41.9–55.2). Current smokers from the aged 16–17 years were 7.5% more than their counterparts aged 13–15 years to obtain cigarettes from commercial sources (62.7% vs 55.2%). In addition, 85.4% of frequent smokers who purchased cigarettes from a commercial store, were almost 1.5 times compared to occasional smokers (85.4%, 95% CI: 77.0–89.4; and 43.7%, 95% CI: 38.3–49.3, respectively; p<0.001) (Table 3, and Supplementary file Figures 1 and 2–8). Multivariable logistic regression (Table 4) showed that males who are current smoke (AOR=1.82; 95% CI: 1.13–2.92), those who were aged 16–17 years (AOR=1.73, 95% CI: 1.27–2.36), frequent smokers (AOR=4.80; 95% CI: 3.11–7.42), adolescents of Indian or Malay descent (AOR=4.76; 95% CI: 1.36–16.73; and AOR=2.11; 95% CI: 1.03–4.10) and current smokers with parents who smoked (AOR=1.50; 95% CI: 1.10–2.25) were more likely to obtain cigarettes from commercial sources. Interaction analysis revealed that only age groups and frequent smoking status are significant (Supplementary file Figure 3). More detailed analysis of the interaction revealed that it occurred between non-frequent smokers aged 13–15 years (49.7%, 95% CI: 44.5–55.0) and respondents aged 16–17 years (60.2%, 95% CI: 55.0–65.2). Whilst more than 9 in 10 frequent smokers of the older age group obtained cigarettes from commercial sources (93.3%, 95% CI: 87.9–96.4) compared to the frequent smokers aged 13–15 years (70.7%, 95% CI: 59.6–79.8).

**Table 3:** Source of cigarettes of current smokers among school-going adolescents by sociodemographic variables, parental smoking status, and frequent smoking status among participants in the National Health and Morbidity Survey 2022: Adolescent Health Survey (NHMS 2022: AHS) (N=1934)

*Variable*	*Commercial source*				*Social source*				*p*
	*Estimated population*	*Sample*	*%*	*95% CI*	*Estimated population*	*Sample*	*%*	*95% CI*	
**Gender**									
Overall	76312	1168	59.7	57.0–62.4	51474	765	40.3	37.6–43.0	
Male	67287	1010	61.6	59.6–64.5	41902	616	38.4	35.5–41.4	<0.001
Female	9025	158	48.5	41.9–55.2	9571	147	51.5	44.8–58.1	
**Ethnicity**									
Malay	51967	832	61.1	57.8–64.4	33023	536	38.9	35.6–42.2	0.001
Chinese	4028	58	47.7	36.8–58.8	4418	48	52.3	41.2–63.2	
Indian	5429	77	79.3	66.9–87.9	1418	16	20.7	12.1–33.1	
Bumiputra Sabah	5264	90	54.6	44.6–64.3	4375	60	45.4	35.7–55.4	
Bumiputra Sarawak	7305	76	55.6	46.9–64.1	5825	64	44.4	35.9–53.1	
Other	2316	35	49.0	35.1–63.0	2413	41	51.0	37.0–64.9	
**Age** (years)									
13–15	37392	537	55.7	51.9–59.5	29686	405	44.3	40.5–48.1	0.002
16–17	38920	631	64.1	60.3–67.8	21787	360	35.9	32.2–39.7	
**Parental smoking**									
Yes	45709	661	62.7	59.1–66.3	27151	405	37.3	33.7–40.9	0.012
No	23483	398	55.2	50.6–59.8	19054	281	44.8	40.2–49.4	
**Frequent smoker**									
Yes	17850	284	85.4	80.2–89.5	3041	55	14.6	10.5–19.8	<0.001
No	38312	577	54.9	51.1–58.6	31523	462	45.1	41.4–58.9	
**Parental marital status**									
Married	57131	885	59.3	56.1–62.3	39280	589	40.7	37.7–43.9	0.847
Separated	16312	237	59.5	53.7–65.8	10907	157	40.5	34.2–46.3	

The estimated population and % (95% CI) were calculated based on a weighted sample based on survey’s sampling design, response rate, and population characteristics to ensure valid population estimates from the analysis.

**Table 4:** Multivariable logistic regression analysis of source of commercial source of cigarettes among school going adolescents, by sociodemographic variables, parental smoking status, and frequent smoking status among participants in the National Health and Morbidity Survey 2022: Adolescent Health Survey (NHMS 2022: AHS)

*Variable*	*AOR*	*95% CI*
**Gender**		
Male	1.82	1.13–2.92
Female ®	1	
**Ethnicity**		
Malay	2.11	1.03–4.10
Chinese ®	1	
Indian	4.76	1.36–16.73
Bumiputra Sabah	2.32	0.99–5.45
Bumiputra Sarawak	1.85	0.82–4.20
Other	1.83	0.68–4.90
**Age** (years)		
13–15 ®	1	
16–17	1.73	1.27–2.36
**Parental smoking**		
Yes	1.50	1.10–2.06
No ®	1	
**Frequent smoker**		
Yes	4.80	3.11–7.42
No ®	1	

AOR: adjusted odds ratio. The estimated population and % (95% CI) were calculated based on a weighted sample based on survey’s sampling design, response rate, and population characteristics to ensure valid population estimates from the analysis. All variables in the model have p≤0.25 in bivariate analysis. These variables were included in the MLR to identify factors related to the sources of cigarettes. ® Reference categories.

## DISCUSSION

The study showed that nearly 60% of current smokers acquired cigarettes from commercial sources. The prevalence is almost 5% higher than the previous national survey using Tobacco and Electronic Cigarettes among Malaysian Adolescents data 2016. The prevalence reported is also higher than the global point three (140 countries) prevalence of (51.2%) of adolescent smokers who bought cigarettes from commercial vendors^14^. In addition, it is more than two times higher than youth smokers in Korea (25.7%)^21^. However, the current prevalence is similar to 62.5% among youth smokers in Tunisia^22^, 25% lower than 83.3% from the GYTS study in China (83.0% of males and 85.2% of females)^15^ and Thailand (67.4%; 95% CI: 58.5–75.3)^16^. In addition, the prevalence was lower than that of most countries in the study by D’Angelo et al.^23^. Compared to the study results in 2016, an increase in adolescents who obtained cigarettes from commercial sources is observed among all variables under investigation. Although the increase in the prevalence of obtaining tobacco products from commercial sources is not significant compared to 2016, it is a concern considering that the regulation preventing the sale of tobacco products to individuals under the age of 18 years has been enforced for almost two decades. The majority of sales premises display signs prohibiting the sale of tobacco products to individuals under the age of 18 years, but it is ignored considering that enforcement is not strict due to the lack of human resources to conduct enforcement activities on premises, which include petrol kiosks, grocery shops, supermarkets, and others that widely sell tobacco products in Malaysia. Studies showed that policies prohibiting adolescent access to cigarettes will not be effective with strict enforcement^13,24^.

The prevalence of purchased tobacco products from commercial sources is higher among male current smokers, of whom >60% obtained tobacco products from commercial sources compared to 48.5% among current female smokers. This finding is in line with the findings of Sun et al.^14^ on the method of obtaining tobacco products among teenagers aged 13–15 years in 140 countries (55.3%), who found that the majority of countries showed a high prevalence among male adolescent smokers who obtained tobacco products from commercial sources, similar findings were also reported by Chotbenjamaporn et al.^16^ and Anh et al.^12^ among adolescents in Thailand and Vietnam, respectively. However, this finding contradicts the results of Lim et al.^7^, which reported that teenage female smokers in urban areas are more likely to get cigarettes from commercial sources. These findings may be due to changes in the dynamics of the acquisition of cigarette sources among teenage smokers over the past six years. This finding may be due to society’s tolerance (permissible social norm), which is higher towards the practice of smoking among male teenagers compared to female teenagers, resulting in the acquisition of cigarettes to be more accessible among teenage boys^18,19,22,23,25^. These studies reported that male adolescents were more likely to acquire cigarettes from commercial sources, which is similar to the outcome of this study. The reluctance of females to smoke often results in their hesitancy to purchase tobacco products from commercial outlets, especially in Malaysian and other Asian societies that follow the principle of communitarianism. This philosophy emphasizes the welfare of the community and the common good over personal desires. It fosters a strong sense of community and social unity, where individuals place the needs of the group ahead of their ambitions.

Communitarianism prioritizes the well-being of society over individual autonomy, which leads individuals to acquire cigarettes from social sources. This value also affects the attitude of cigarette sellers. Chotbenjamaporn et al.^16^ found that over 60% of female smokers were denied cigarettes by retailers. Compared to only 40% of male adolescent smokers, even though female teenagers may be more mature than male smokers, societal norms and retailer attitudes may hinder them from obtaining cigarettes from commercial sources. In addition, it is customary in Malaysia for parents or guardians to prioritize the needs of female adolescents. In contrast, male adolescents, who are expected to be future family leaders, are granted greater freedom to explore their surroundings^17^. This independence may increase the likelihood of adolescent boys acquiring tobacco by purchasing it. However, this hypothesis needs to be tested in future studies. Also, parents in Malaysia pay more attention to their daughters compared to their sons; the freedom and space that male smokers get, makes it easier for them to get cigarettes from commercial sources^17,18^.

Fixed premises such as grocery stores, petrol kiosks etc., are the primary commercial sources of cigarettes, and friends are the primary social sources. This finding is almost identical to the findings reported by Lim et al.^7^ six years ago. Similar findings were also reported by Chotbenjamaporn et al.^16^ among Thai adolescent smokers; furthermore, Chen et al.^26^ reported that almost a third of Taiwanese youth smokers also obtain cigarette products from a similar source. The study also showed that more than 1 in 10 respondents gets it from non-fixed premises, indicating that tobacco products are available in every locality niche in Malaysia and are easy to obtain. The absence of a detection mechanism, such as licensing of premises, creates difficulties in detecting the cause of the acquisition of tobacco products among teenagers. The study also found that only a small number of teenagers obtain tobacco products at food stores. We postulated that teenagers are less likely to visit these premises to get tobacco products, considering that the number of customers is more significant at food eateries compared to other fixed premises and the likelihood of meeting known individuals is higher compared to other premises such as retail shops and supermarkets. Perceived harmful social norms among smokers cause them to look for safer alternatives. In addition, this study found that adolescents have begun to buy tobacco products online; this is a challenge faced with the increasingly difficult to implement control methods. A mechanism to regulate the purchase of tobacco products online should be established to curb tobacco products through this source.

In line with prior studies^7,12,15^, our study has shown a significant association between age group and tobacco products obtained from commercial sources. At the same time, social access tends to diminish with age. In adolescents, physical and psychological development progress significantly, which causes them to behave more like adults; this allows them to obtain tobacco products more efficiently than their counterparts who are aged 13–15 years. In addition, teenagers in the older age group are usually given more freedom and more pocket money by their parents/guardians, considering that financial needs increase according to age. In addition, teenagers in this age group are more mature and skilled in communication, making it easier to obtain tobacco products from commercial sources^7^. In addition, older adolescents may have more significant financial means to purchase cigarettes through commercial channels, as high school students in their later years may have part-time employment and hence have the ability to finance over-the-counter transactions.

After adjusting for confounding factors, our study found that individuals who are frequent smokers were more inclined to buy their cigarettes. Other studies also revealed similar findings, where frequent smokers usually get tobacco from various sources to meet their needs. The results of frequent smokers getting tobacco are also the highest of all the independent variables investigated in this study, which are the same as the findings reported by Lim et al.^7^ in their study 6 years ago. Frequent smokers often recognize typical retail establishments that provide convenient availability of cigarettes, such as supermarkets and convenience stores. As regular purchasers, they may be acquainted with specific locations where they can obtain cigarettes through unlawful means. Regular smokers may also frequent the same establishment to purchase cigarettes, thus developing a strong rapport with the business owner and obtaining cigarettes more conveniently from that particular store. In addition, individuals who smoke regularly are more prone to purchasing cigarettes from retail establishments as a result of their tobacco addiction. Addiction is a dynamic phenomenon that usually starts with sporadic smoking and eventually advances to frequent smoking. Smokers who smoke often encounter challenges when attempting to quit, due to their existing addiction to tobacco^25^. The more advanced stages of smoking are strongly linked to the act of buying their final cigarette. Many young people start buying their cigarettes if they become dependent on them due to higher levels of cigarette consumption.

This study found that 8 out of 10 teenage smokers of Indian descent were more likely to obtain cigarettes from commercial sources. This finding is at odds with the findings reported by Lim et al.^7^ who noted that Malay adolescent current smokers are the largest group that obtains tobacco products rather than commercial products. An increase of >30% in the acquisition of tobacco products among those of Indian descent is something surprising, considering that adolescents from ethnic groups show a smoking prevalence that is somewhat similar to that of Chinese adolescents in obtaining tobacco products^7^. This rather drastic change requires further and in-depth study among ethnic Indian adolescent smokers, to get a clearer picture of how drastic the increase is among them. Quantitative and qualitative analyses are necessary to summarize this study’s findings.

This study found that current smokers with parents or guardians who smoke are more likely to source their tobacco from commercial sources. This finding is interesting, considering that current smokers prefer to get cigarettes from parents or guardians who are close to them. However, they are more likely to obtain tobacco products from commercial sources. This finding may be due to adolescent smokers perceiving that their parents/guardians have negative attitudes and respond to the practice of smoking among their children even though they are smokers, which has been reported by Lim et al. in their studies among school-going adolescents in Kota Tinggi in Johor^17^ and Petaling District in Selangor^18^. In addition, Malaysia is a community that prioritizes respect for parents; they always follow the perception or attitude of parents/guardians^27,28^, including the behavior they practice. Smokers may drive this respect to obtain tobacco products that will not undermine respectful family relationships.

### Limitations

This study has some limitations. Initially, we omitted an evaluation of the shopkeepers’ understanding and opinions concerning the laws forbidding selling tobacco to anyone <18 years. Secondly, the data on cigarette sources rely on self-reporting, which introduces the possibility of respondents providing inaccurate or incomplete information. Thirdly, the cross-sectional study only enables us to measure association and cannot attribute causality. Fourthly, there is the possibility of response and recall bias and the possibility of residual confounding. Lastly, the results only enable generalizability to Malaysian school-going adolescents and may not be applicable to school-going adolescents in other countries.

### Implications

Our research indicates that the tobacco control methods proposed by the WHO FCTC^5^ should be enhanced. Supply-side measures are especially pertinent when it comes to preventing minors from purchasing cigarettes. Robust measures to address the illegal trade of tobacco, prohibiting the sale of tobacco to individuals under the legal age, increasing the minimum age for selling cigarettes to individuals up to 21 years old, requiring cigarette retailers to verify the age of customers, and restricting the sale of tobacco products to licensed retailers only as a licensing system for tobacco outlets, could prove advantageous. Implementing a licensing system for selling cigarettes could be an effective regulatory mechanism. Under this system, all tobacco shops must obtain a license to sell tobacco products. This proposal would empower the government to enhance its monitoring of young people’s ability to get tobacco, exposure to advertising, concentration of tobacco sellers, and location of tobacco stores. Within this licensing framework, licensees must authenticate the age of a cigarette purchaser by checking their identity and age, preventing any infringement of regulations. Moreover, it is imperative to bolster health promotion programs to augment shop awareness and comprehension of the existing legislation that forbids the sale of cigarettes to underage individuals^12-14,25^ in addition to empowering the retailer to decrease tobacco sales to consumers^27^. The empowering process should include establishing partnerships, active listening, engaging in discourse, reflecting on experiences, taking action, and providing feedback to raise their self-awareness. These measures will generate dynamic dialogues where the retailer can receive support in recognizing and elucidating personal challenges and resolutions by harnessing their strengths and capacities, to effectively protect the health of adolescents and preserve a healthy environment.

## CONCLUSIONS

The NHMS 2022: Adolescent Health Survey reveals that most (59.7%) Malaysian youth smokers obtain cigarettes from commercial sources, with higher prevalence among frequent smokers, males, older adolescents, and those with smoking parents. Despite regulations, weak enforcement allows easy access to cigarettes. Strengthening law enforcement, retailer compliance, and stricter measures like licensing and age verification can help curb youth smoking. Public health campaigns, community engagement, and further research on retailer attitudes and online cigarette sales, may be crucial to enhancing tobacco control and protecting public health.

## Supplementary Material



## Data Availability

The data supporting this research are available from the authors on reasonable request.
