# Supplementary material for: Sources of cigarettes for youth smokers in Malaysia: Findings from the National Health and Morbidity Survey (NHMS) 2022: Adolescents Health Survey (AHS)
Source: Tob Induc Dis. 2025 Mar 24;23:10.18332/tid/201987. doi: 10.18332/tid/201987 (PMC11931627; doi:10.18332/tid/201987)
Supplement: Supplementary file 1 [file TID-23-39-s1.pdf]

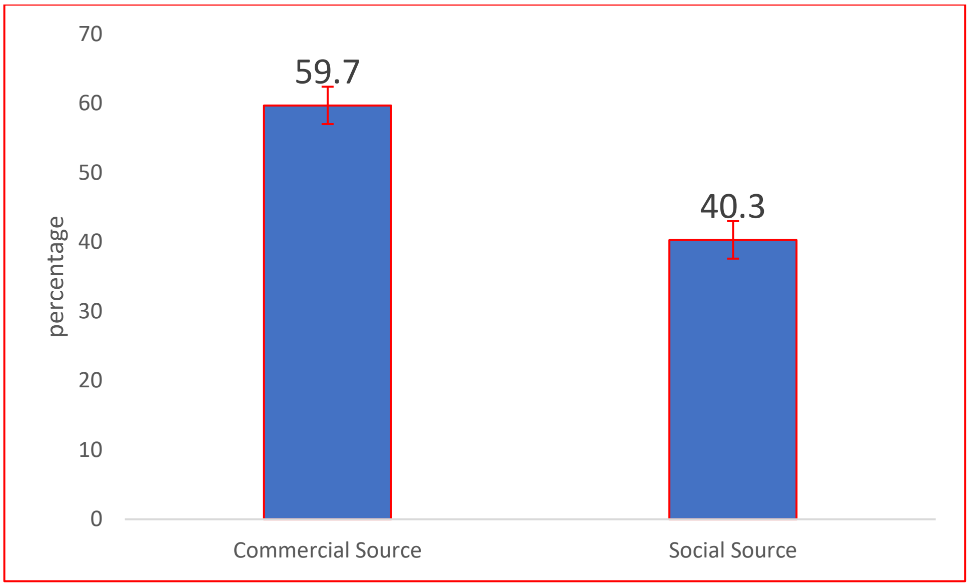

Figure 1(a): Percentage of source of cigarette among current cigarette smokers secondary school adolescents participated in NHMS: AHS 2022

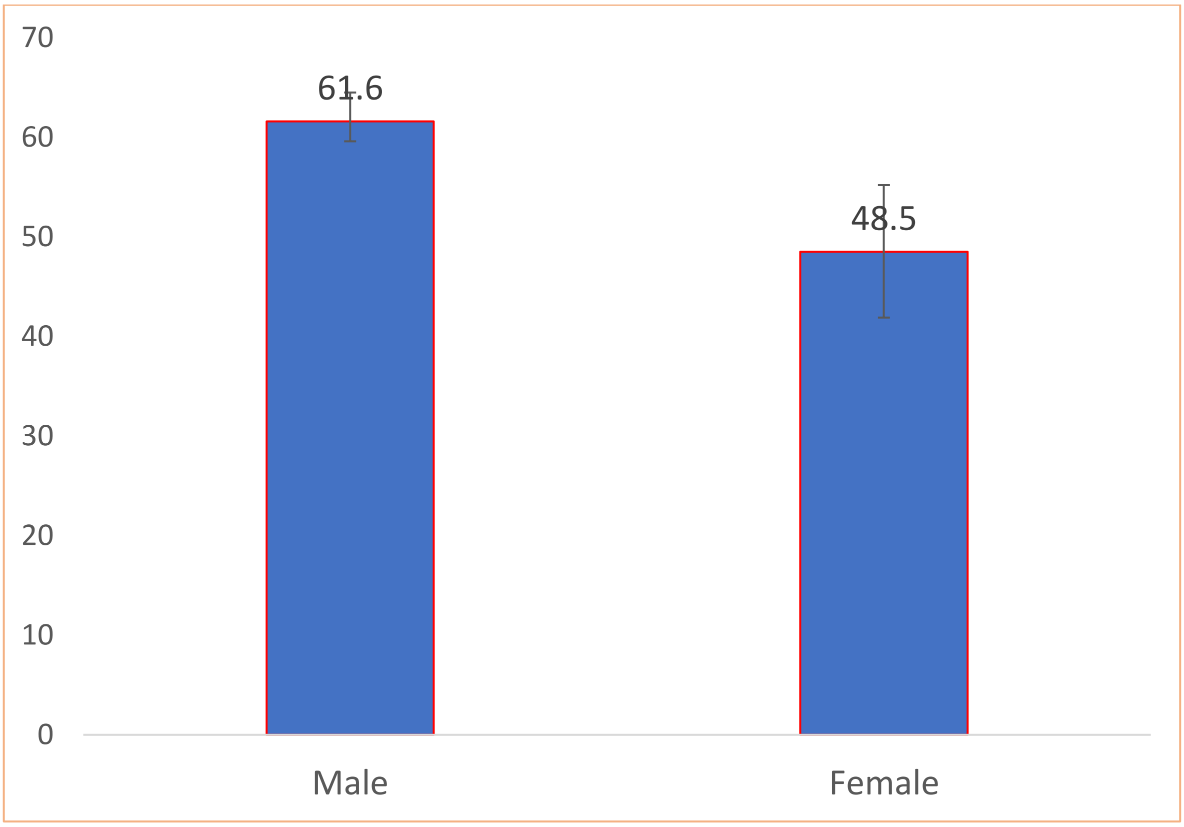

Figure 2(a): Percentage of current cigarette smoker obtained the cigarette from the commercial source by gender among secondary school-going adolescents who participated in the NHMS: AHS 2022

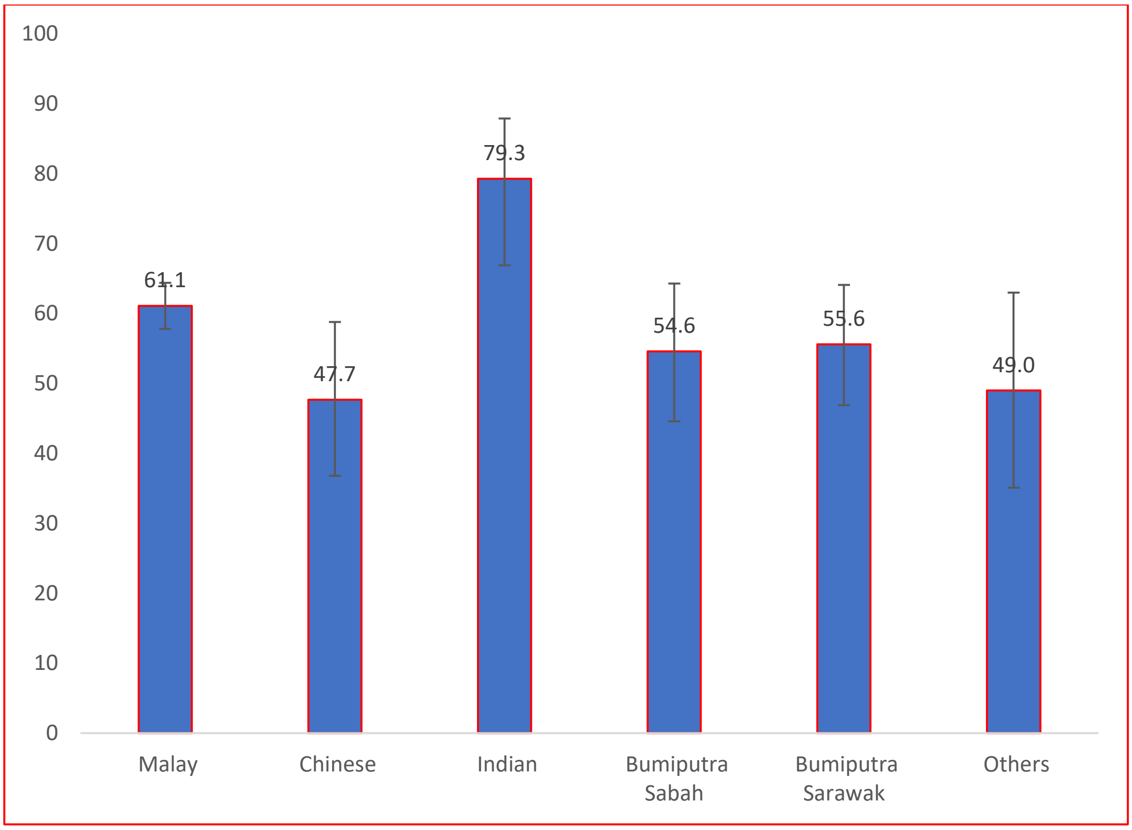

Figure 2(b): Percentage of current cigarette smoker obtained the cigarette from the commercial source by ethnicity among secondary school-going participated in the NHMS: AHS 2022 study

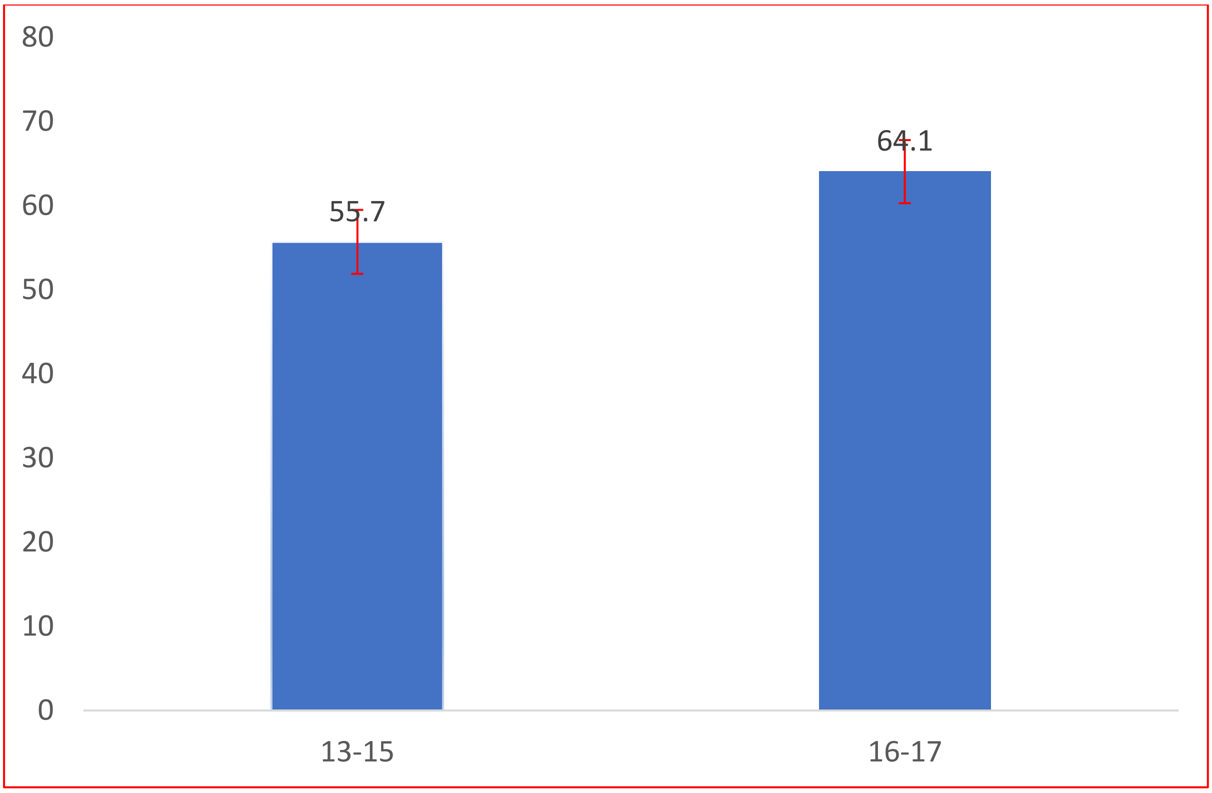

Figure 2(c): Percentage of current cigarette smoker obtained the cigarette from the commercial source by age-groups among secondary school-going participated in the NHMS: AHS 2022 study

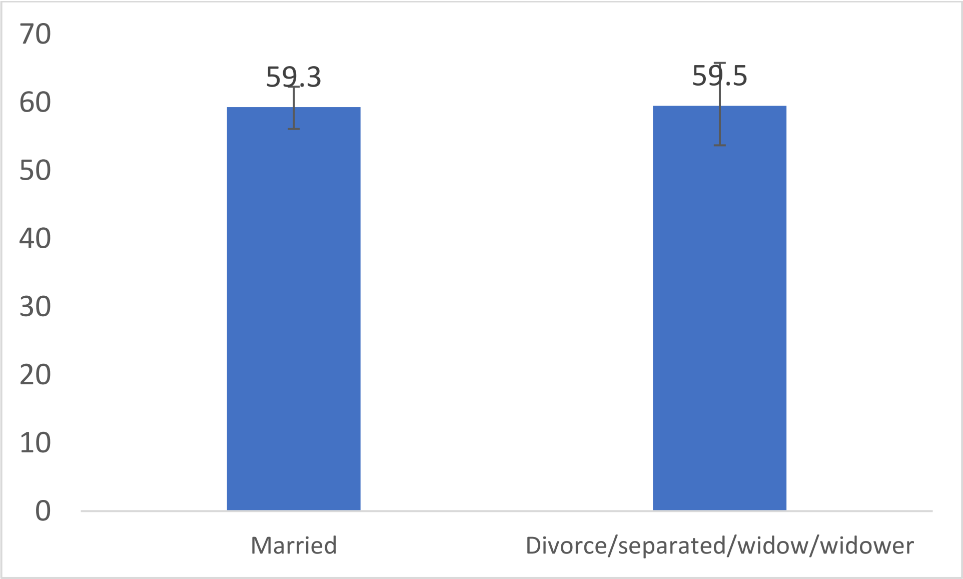

Figure 2(d): Percentage of current cigarette smoker obtained the cigarette from the commercial source by parental marital status among secondary school-going participated in the NHMS: AHS 2022 study

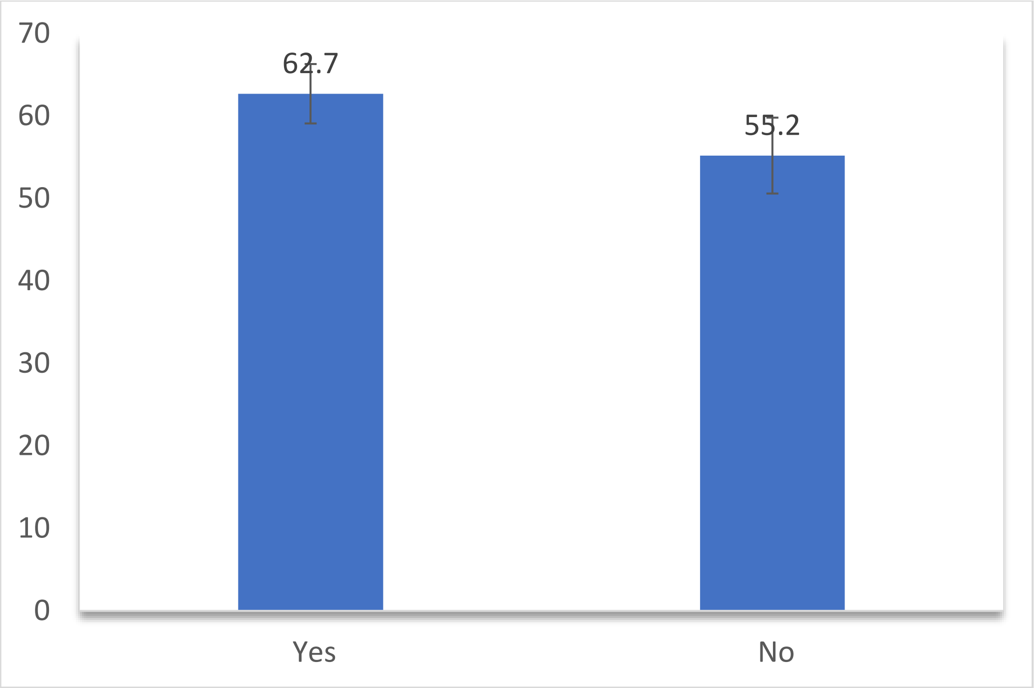

Figure 2(e): Percentage of current cigarette smoker obtained the cigarette from the commercial source by parental smoking status among secondary school-going participated in the NHMS: AHS 2022 study

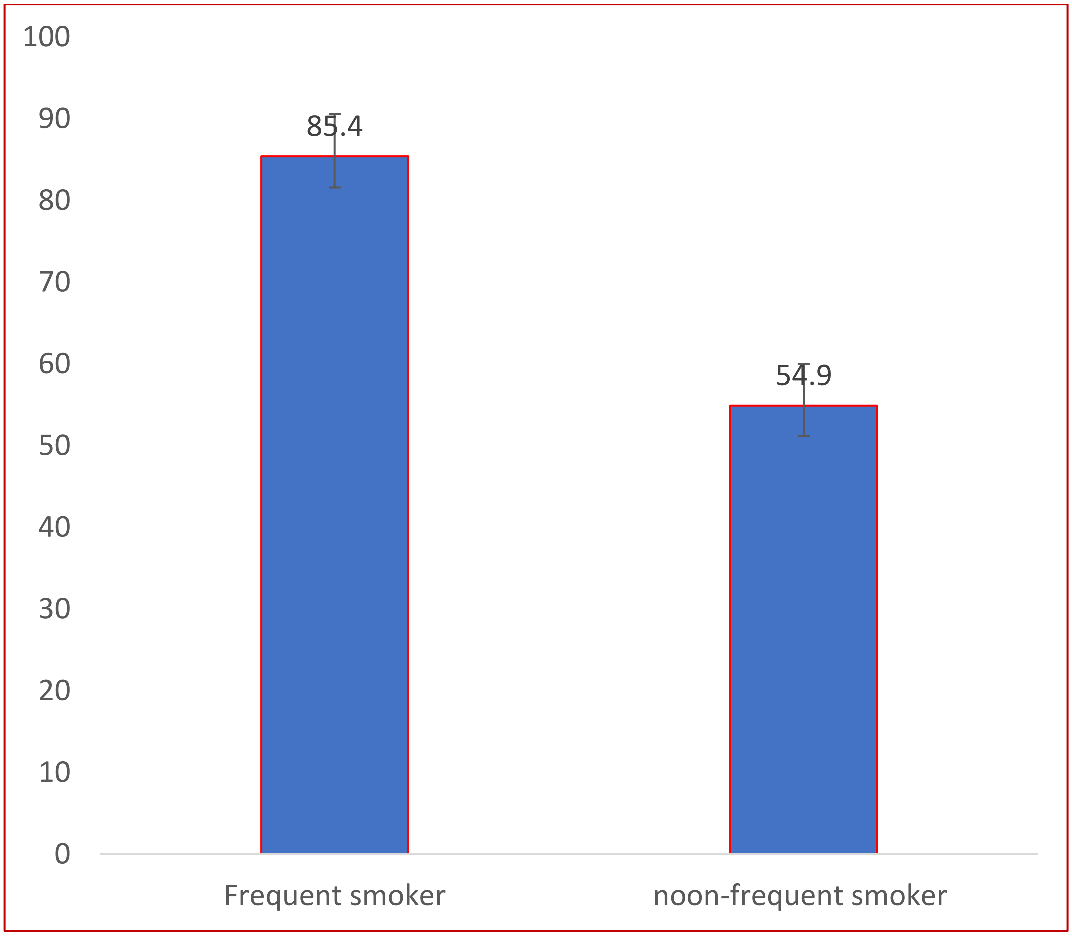

Figure 2(f): Percentage of current cigarette smoker obtained the cigarette from the commercial source by frequent smoker status among secondary school-going participated in the NHMS: AHS 2022 study

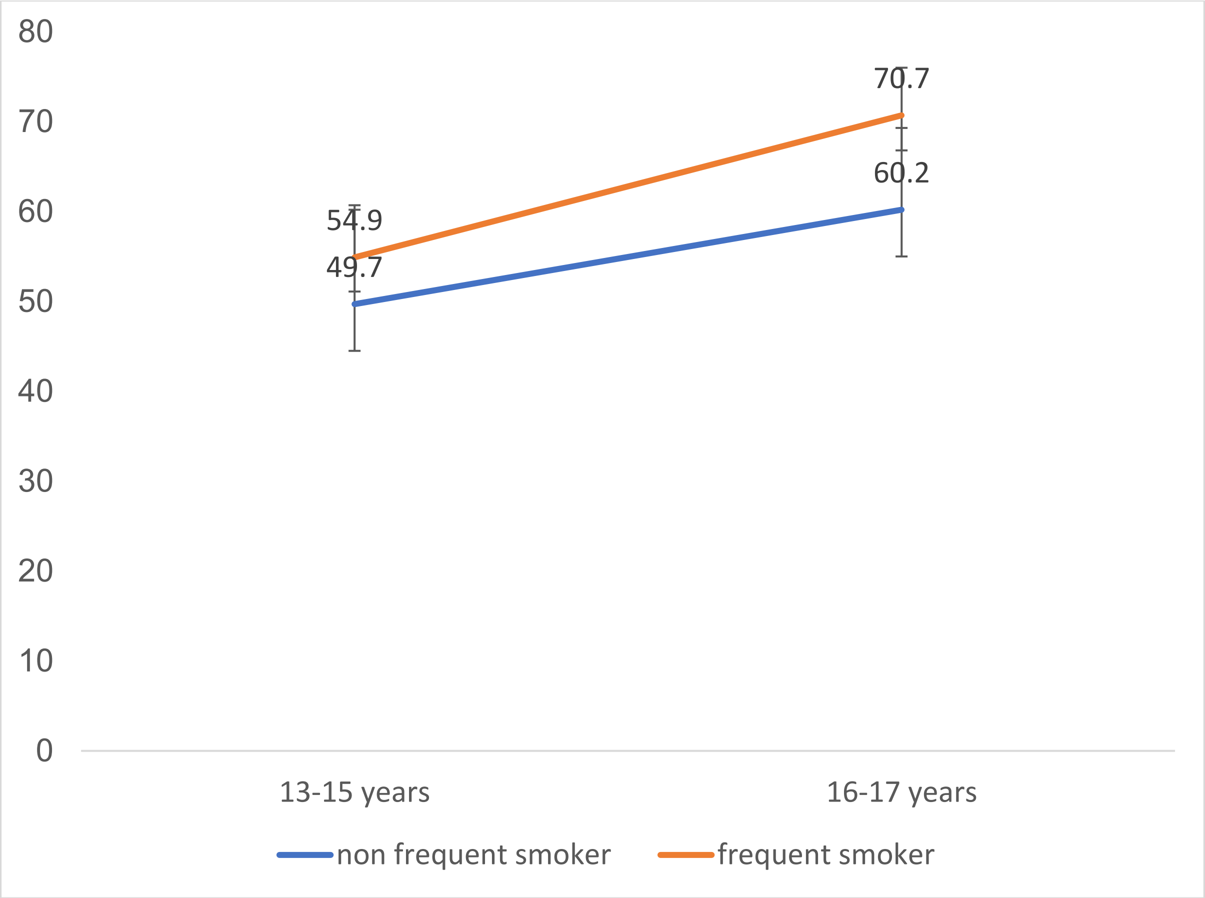

Figure 3: Interaction between age-group and frequent smoking status among current cigarette smoker who participated in NHMS: AHS 2022 study.

© 2025 Lim K.H. et al.
